# ‘I need time to start antiretroviral therapy’: understanding reasons for delayed ART initiation among people diagnosed with HIV in Lusaka, Zambia’

**DOI:** 10.1080/07853890.2022.2051069

**Published:** 2022-03-21

**Authors:** Chanda Mwamba, Laura K. Beres, Stephanie M. Topp, Njekwa Mukamba, Sandra Simbeza, Kombatende Sikombe, Aaloke Mody, Elvin Geng, Charles B. Holmes, Caitlin E. Kennedy, Izukanji Sikazwe, Julie A. Denison, Carolyn Bolton Moore

**Affiliations:** a Centre for Infectious Disease Research in Zambia, Lusaka, Zambia; b Department of International Health, Johns Hopkins Bloomberg School of Public Health, Baltimore, MD, USA; c College of Public Health, Medical & Veterinary Sciences, James Cook University, Townsville, Australia; d Department of Public Health Environments and Society, Faculty of Public Health and Policy, London School of Hygiene and Tropical Medicine, London, United Kingdom; e Washington University School of Medicine in St. Louis, MO, USA; f Georgetown University, Washington, DC, USA

**Keywords:** ART initiation, delayed treatment, antiretroviral refusal, test and start, Zambia

## Abstract

**Introduction:**

Rapid antiretroviral therapy (ART) initiation can improve patient outcomes such as viral suppression and prevent new infections. However, not everyone who can start ART does so immediately.

**Methods:**

We conducted a qualitative study to inform interventions supporting rapid initiation in the ‘Test and Start’ era. We purposively sampled 20 adult patients living with HIV and a previous gap in care from ten health facilities in Lusaka, Zambia for interviews. We inductively analysed transcripts using a thematic, narrative approach. In their narratives, seven participants discussed delaying ART initiation.

**Results:**

Drawing on messages gleaned from facility-based counselling and community information, many cited greater fear of rapid sickness or death due to imperfect adherence or treatment side effects than negative health consequences due to delayed initiation. Participants described needing time to ‘prepare’ their minds for a lifetime treatment commitment. Concerns about inadvertent HIV status disclosure during drug collection discouraged immediate initiation, as did feeling healthy, and worries about the impact of ART initiation on relationship dynamics.

**Conclusion:**

Findings suggest that counselling messages should accurately communicate treatment risks, without perpetuating fear-based narratives about HIV. Identifying and managing patient-specific concerns and reasons for the ‘need for time’ may be important for supporting individuals to rapidly accept lifelong treatment.

**Key messages**
1. Fear-based adherence messaging in health facilities about the dangers of missing a treatment dose or changing the time when ART is taken contributes to Zambian patients’ refusals of immediate ART initiation
2. Responsive health systems that balance a stated need for time to accept one's diagnosis and prepare to embark on a lifelong treatment plan with interventions to identify and manage patient-specific treatment related fears and concerns may support more rapid ART initiation
3. Perceived social stigma around HIV continues to be a significant challenge for treatment initiation.

## Introduction

Despite improved patient outcomes with rapid antiretroviral therapy (ART) initiation [1–4] and widespread adoption of the World Health Organization’s ‘test and start’ guideline [5], many people living with HIV delay ART initiation [6–11]. A clear understanding of the reasons and factors influencing delayed ART is needed to inform effective interventions for more rapid initiation [2,4]. Like many sub-Saharan African countries, Zambia adopted the ‘test and start’ strategy in 2016 [12,13]. However, treatment initiation remains sub-optimal [11,14].

Multiple individual, psychosocial, structural, and health system-related factors are known to influence ART initiation [15–19]. A systematic literature review of studies prior to ‘test and start’ in sub-Saharan Africa, including two studies from Zambia, identified barriers to ART initiation including fear of stigma, disclosure, and side effects; competing livelihood commitments; structural barriers (e.g. distance and costs); and clinic-related barriers such as long queues and poor health care worker (HCW) attitudes [20–24]. Since ‘test and start’ began, four quantitative studies in Zambia [25–28] and qualitative studies from Swaziland and Mozambique identified barriers similar to the pre- ‘test and start’ era, with additions including difficulties accepting diagnosis [29–31]. Most of the extant literature on ART initiation in Zambia and the sub-Saharan African region is quantitative. Among published qualitative studies, only one, from Tanzania [32], utilises narrative methods. Narrative research allows for identification of meaning and sense-making from the perspective of the person relating the story [33–37]. This patient-centered [38] perspective is critical to understand and address drivers of delayed ART initiation. Our study sought to understand reasons for delayed ART start from the patient perspective and their interpretation of delayed ART experiences to inform interventions to optimise treatment outcomes in Zambia.

## Methods

### Study background and population

Participants were sampled from a larger study that identified vital status and care outcomes among a probability sample of adult patients considered lost to follow-up from HIV care in Zambia [39,40]. Qualitative interviews were conducted between January and June 2019 with a subset of participants from Lusaka Province who self-identified as disengaged from care, but subsequently re-engaged [41]. Out of 65 eligible participants with telephone contact details in their medical files, 51 had working telephone numbers. After ≥3 call attempts to all 51, we successfully contacted 27, of whom 4 had died, 3 declined, and 20 consented to participate. Among the 20 participants, seven narrated experiences of delaying ART during the broader narrative of their HIV care journey. Since it was not asked about directly, understanding delayed ART among the subset of participants who raised it as a noteworthy component of their HIV care narrative offers unique and important insight into the behaviour that can guide intervention development. This paper presents the results of a secondary data analysis of the sub-group of participants who discussed delaying ART initiation in their interviews.

### Procedures

Participants were recruited and interviewed in person by the lead author (CM) following written informed consent. Interviews were conducted in English, Nyanja or Bemba, per patient preference and lasted 1–2 h. Using an individualised guide designed to elicit a patient’s narrative of care engagement, participants narrated their experience of their HIV care and treatment journey since their HIV diagnosis. Participants were not directly questioned about reasons for delayed ART initiation. The narrative approach allowed for patient-led sharing of events and contexts associated with delayed ART start, and the meaning attached to these experiences [33–37].

### Analysis

Interview audio recordings were simultaneously translated into English and transcribed verbatim. After each interview, an analysis memo was created within 24 h and LB and CM discussed the memos and their meanings. Based on the interview scripts, memos and dialogue, we created chronological narratives of each patient’s care journey describing contexts and events that influenced their disengagement, re-engagement and any subsequent delays with ART initiation. Each patient summary and transcript was iteratively read in detail, inductively coded using narrative thematic analysis and categorised into themes [33–37]. Analysis and interpretation of ART-delay related themes was restricted to narratives mentioning delayed ART initiation. Analysis was conducted by LB and CM, including dialogue throughout the stages of the analysis process.

### Results

Seven participants from seven health facilities, aged 21–50 years, and all-female but one, described delayed ART initiation. They were recommended to start ART and initiated between 2013 and 2017. While the exact length of treatment delay was not captured, narratives suggested delays of 3–12 months. Other elements of patient journeys, including entry into care, time from entry to recommended initation, and disengagement were varied across the seven participants [42]. Key themes are described below. Supporting quotes are summarised in Table 1.

**Table 1.** Themes and quotes.

Theme	Quote
‘If you skip treatment you will die’: Misconceptions and misinformation	At the clinic where we were collecting drugs, they were saying that you don’t need to miss taking drugs and don’t exceed the allocated time for taking drugs, like if you are taking drugs at twenty-one hours then you take at twenty-three hours, this means that you are creating your own problems, because of that I was getting scared knowing that sometimes one may forget or one may be busy at the time they take medicine such that by the time you take the drugs maybe some minutes would have passed, so I was worried that this will be our death now. [Female, 35–44] (Friends) said that when you skip the treatment you will die, while others said when you forget to take them you will die (….), so those who have not yet started, they tend to be scared [Female, 55+] Okay, what happens when you start then you stop, (…) it’s like you’re giving chance to that virus to come with full force (…) And when it comes with full force a lot of illnesses keep coming in. So that’s what I didn’t want because with me I felt it’s better for me like this without such complications, it will be better than starting something which I won’t manage to finish by the end of the day. [Male, 25–34]
‘I need to think’: Preparing for a life-long commitment	I said I need to think about it because you know, it’s not something easy whereby you just wake up and say let me start this. That’s why sometimes you find that people start then they fail to continue or stop. (Female 34–44) At first, because I was scared, thinking … how will I start…at which facility will I start from, so you think of all that, that’s why I was holding back to say I will start taking drugs but needed them (HCWs) to wait for me first. (Female 18–24)
‘I don’t want people to know my status’: Concerns about HIV disclosure	that issue of screaming [calling out names of people waiting for ART at the clinic] affects me because I don’t want people to know my status, I don’t want them to know, for me I don’t! maybe I might get depressed and stop my medicine and that is something I don’t want to do. [Female, 18–24] I could get embarrassed because if you meet someone you know at the clinic then they could tell someone they know, they could be talking in the community that we have found such a person collecting drugs at the clinic, so that is what I was scared of [Female, 35–44]
‘I was feeling well with my health’: Difficulty accepting HIV Diagnosis	‘So, when I went there (VCT), I was told, you are HIV positive, then I said hmm how come? I could not believe it because I was feeling well with my health. Then from there, I went to another clinic to test again’ [Female 45+] R: I didn’t feel good [about diagnosis], and the reason I didn’t feel good is that I looked at my life and what I went through, how I looked after myself…I had not had multiple partners [Female, 25–34] You know the way we look at HIV… [made accepting my HIV status] quite challenging, it was something scary because now I was thinking so I will die soon! Then even when you know the fact that you can’t die if you take your medication and accept your status and you can live long, there is just always that fear, it will just pop into your mind. [Female, 25–34 years]
‘How am I going to manage’: Concerns about ART consumption	By then, people would talk that the drugs, the tablets are big and then I thought about it, because already I have a problem taking drugs like Panadol, I usually break it, how about ARVs how am I going to manage them? [Female, 35–44] because I’ve seen some side effects from some other people I don’t know, you find that others start developing a big tummy or maybe the chest is too big (….). You can even tell just from the way they look that they are on treatment. Yes so I was scared of such side effects so to me it was like a gamble because I didn’t know if I’ll have the same side effects or not. [Female, 25–34]

### ‘If you skip treatment you will die’: Misconceptions and misinformation

Participants reported receiving counselling messages that emphasised the importance of perfect adherence, rather than the overall benefits of treatment. These post-test messages from HCWs were reinforced through information heard in participants’ communities. Participants described understanding that delaying treatment was better than starting if they could not guarantee that they would take ART at the same time, daily, for a lifetime. They feared that skipping a dose or stopping for any reason would ‘bring a lot of illnesses with full force’, likely causing death more quickly than if they had not initiated.

### ‘I need to think’: Preparing for a life-long commitment

When a HCW recommended ART, some participants explicitly asked to delay immediate start and return after they had time to ‘prepare’ themselves to commit to a lifetime of treatment. Preparation was considered necessary to avoid defaulting. One female participant ‘negotiated’ with the HCW for 3 months before starting ART, stating she needed the time to think about a ‘lot of things’ as starting ART is ‘not something easy whereby you just wake up and say let me start this.’ Preparation time also involved planning treatment access, and reflection on dealing with potential unintentional status disclosure.

### ‘I don’t want people to know my status’: Concerns about HIV disclosure

Worries about inadvertent status disclosure and social consequences of disclosure also discouraged initiation. Participants cited crowded health facilities and the ongoing practice of ‘screaming’ out ART patient names when it was their turn to see a clinician as threatening disclosure, leading to ‘being gossiped about’, ‘embarrassment’, ‘shame’, and ‘depression’. Social relationship dynamics modulated disclosure fears, with emphasis on how family and friends would think of them henceforth. One married participant’s fear of being blamed for infecting her husband with HIV led her to seek re-testing as a couple before initiation.

### ‘I was feeling well with my health’: Difficulty accepting HIV diagnosis

Many participants described initial difficulty accepting their HIV diagnosis, and associated fear of HIV and its consequences, delaying ART start. Participants said they tested repeatedly at different clinics to verify results and felt ‘lazy’ to start treatment if they considered themselves ‘healthy’. Having been faithful to a sexual partner compounded difficulties with status acceptance

### ‘How am I going to manage’: Concerns about ART consumption

Concerns about ART, particularly fears of possible side effects and difficulties swallowing large pills delayed initiation. Participant fears were heightened by community narratives about ‘big tablets’, hallucinations, bad dreams, tumours, and body changes. Contrary to fears before starting ART, multiple participants narrated personal experiences of minimal side effects post-initiation.

## Discussion

We identified factors influencing delayed ART initiation among adult, Zambian patients with a history of a gap in HIV care. While adherence remains a key part of the clinical narrative, we found that fear-based messaging from HCWs emphasising the consequences of non-adherence are an important factor in delayed initiation. Our finding highlights the need for clear, positive, and nuanced facility-based communications around ART to assist patients in overcoming feelings of fear and assist them in taking decisive steps to initiate life-long treatment.

Given patients’ need for preparation, targeted improved counselling efforts are likely required to serve the health system goal of rapid initiation. Better counselling may include supporting patients to adjust to initiation of ART then subsequent, follow-ups to support the readiness needed for sustaining ART long-term; options-based messaging such as contingency planning for what to do if a dose is missed (e.g. take immediately, skip until next dose, etc); and/or addressing human resource constraints that limit time and quality of counselling interactions. Improved approaches may also help address how patients’ experiences of good health can motivate ART initiation [15,16,31]. Further, community intervention is needed to clarify ART requirements and benefits for patients and their partners [43–45]. Our findings align with other qualitative studies in the region since the ‘test and start’ era, which identifies the need by some patients for time to emotionally process the diagnosis, come to terms with the new realities of illness and treatment, and weigh the potential social and individual consequences of being on ART [16,28,46–48]. Given extant literature demonstrating an increased risk of loss-to-follow-up after same-day ART initiation (2.5 times the likelihood in one Ugandan study) [49], the identified factors warrant an intentional response to improve rapid initiation [50–53]. Such approaches may include patient-centred approaches [54,55] that offer appropriate follow-up support to retain patients after ART initiation. Outreach and kindness can facilitate patient engagement [56]. Our study reinforces the literature that the social complications associated with disclosure affect myriad HIV-related concerns, including, as shown in our data, delayed initiation of ART [30]. While there have been consistent efforts targeted at couples such as HIV couple counselling and testing [57], there is a need to recognise that HIV initiation is a socially constructed decision [58] with individuals weighing their social situations in decisions to initate on ART. ART programs can address patients’ social circumstances when initiating ART by using patient-centered communication strategies and tools such as differentiated service delivery (DSD) and community-engagement to facilitate more rapid initiation [54,59,60].

## Study limitations

This study inductively identified themes from seven patient narratives of HIV care and treatment among previously disengaged patients. While a small sample size, the use of a narrative approach provided rich, unprompted, information, which reflects participants’ lived experiences. However, the study sample had only one male, and the study was limited to the capital city, which is more urban and wealthier than other areas. Therefore, the study may not have identified the full breadth of reasons for the delay for these other categories. Our sample included all willing participants among patients from the probability-based sample of the parent study who returned to HIV care in Lusaka. The proportion recounting delay was consistent with regional estimates of treatment refusal [17,41].

## Conclusion

Health providers in the ‘test and start’ era must be aware of misperceptions and a “fear narrative” about HIV, its treatment demands, and side effects. It is important then that the facility messaging addresses these fears to reassure patients while giving them comprehensive and accurate information about the benefits and realities of ART. Consistency in reasons for delayed ART initiation across settings warrants a dedicated response to reduce time to initiation. Interventions to appropriately support patients’ need for time while building individual judgement and agency of HIV care, social and health system resilience may shorten their time to initiation.

## Ethical approval and consent to participate

This study was approved by the University of Zambia Research Ethics Committee, the Zambian Ministry of Health, and the University of Alabama at Birmingham Institutional Review Board (UAB IRB). The Johns Hopkins University and the University of California at San Francisco had reliance agreements with the UAB IRB. Written informed consent for participating in the study was sought and granted by all the interviewees who agreed to participate in the study. Permission to record interviews was sought from all the study participants

## Data Availability

'Human subjects’ protection approval for this study was given on condition that the confidentiality of all respondents would be upheld. Therefore, sharing direct interview transcripts which include locations and personal information may identify respondents which would be inappropriate.

## References

[1] UNAIDS. 90-90-90: an ambitious treatment target to help end the AIDS epidemic [WWW Document]; 2014. [cited 3 Oct 2016]. http://www.unaids.org/en/resources/documents/2014/90-90-90.

[2] Grinsztejn B, Hosseinipour MC, Ribaudo HJ, et al. Effects of early versus delayed initiation of antiretroviral treatment on clinical outcomes of HIV-1 infection: results from the phase 3 HPTN 052 randomised controlled trial. Lancet Infect Dis. 2014;14(4):281–290. 24602844 10.1016/S1473-3099(13)70692-3 PMC4144040

[3] Anglaret X, Minga A, Gabillard D, et al. AIDS and Non-AIDS morbidity and mortality across the spectrum of CD4 cell counts in HIV-infected adults before starting antiretroviral therapy in Cote d'Ivoire. Clin Infect Dis. 2012;54(5):714–723. 22173233 10.1093/cid/cir898 PMC3275759

[4] Ford N, Migone C, Calmy A, et al. Benefits and risks of rapid initiation of antiretroviral therapy. AIDS. 2018;32(1):17–23. 29112073 10.1097/QAD.0000000000001671 PMC5732637

[5] WHO. Guideline on when to start antiretroviral therapy and on pre-exposure prophylaxis for HIV. Geneva: World Health Organization; 2015. 26598776

[6] Bor J, Ahmed S, Fox MP, et al. Effect of eliminating CD4-count thresholds on HIV treatment initiation in South Africa: an empirical modelling study. PLOS One. 2017;12(6):e0178249. 28617805 10.1371/journal.pone.0178249 PMC5472329

[7] Fox MP, Rosen S. A new Cascade of HIV care for the era of “treat all. PLOS Med. 2017;14(4):e1002268–11. 28399160 10.1371/journal.pmed.1002268 PMC5388465

[8] Govindasamy D, Ford N, Kranzer K. Risk factors, barriers and facilitators for linkage to antiretroviral therapy care: a systematic review. AIDS. 2012;26(16):2059–2067. 22781227 10.1097/QAD.0b013e3283578b9b

[9] Haber N, Tanser F, Bor J, et al. From HIV infection to therapeutic response: a population-based longitudinal HIV cascade-of-care study in KwaZulu-Natal, South Africa. Lancet HIV. 2017;4(5):e223–e230. 10.1016/S2352-3018(16)30224-7 PMC5964602 28153470

[10] Rosen S, Fox MP. Retention in HIV care between testing and treatment in sub-Saharan Africa: a systematic review. PLOS Med. 2011;8(7):e1001056. 21811403 10.1371/journal.pmed.1001056 PMC3139665

[11] Mugglin C, Estill J, Wandeler G, et al. Loss to programme between HIV diagnosis and initiation of antiretroviral therapy in sub-Saharan Africa: systematic review and meta-analysis. Trop Med Int Health. 2012;17(12):1509–1520. 22994151 10.1111/j.1365-3156.2012.03089.x PMC3895621

[12] MOH. Zambia consolidated guidelines for treatment and prevention of HIV infection. Lusaka, Zambia: Ministry of Health, Republic of Zambia; 2018.

[13] Motsoaledi A. Health Dept Budget Vote Speech 2016/17 [Internet]. Department of Health, South Africa; 2016. http://www.gov.za/speeches/debate-healthbudget-vote-national-assembly-10-may-2016-dr-aaron-motsoaledi-minister-health.

[14] Iwuji CC, Orne-Gliemann J, Larmarange J, et al. Uptake of home-based HIV testing, linkage to care, and community attitudes about ART in rural KwaZulu-Natal, South Africa: descriptive results from the first phase of the ANRS 12249 TasP cluster-randomised trial. PLOS Med. 2016;13(8):e1002107. 27504637 10.1371/journal.pmed.1002107 PMC4978506

[15] Horter S, Thabede Z, Dlamini V, et al. "Life is so easy on ART, once you accept it": acceptance, denial and linkage to HIV care in Shiselweni, Swaziland”:. Soc Sci Med. 2017;176:52–59. 28129547 10.1016/j.socscimed.2017.01.006

[16] Kawuma R, Seeley J, Mupambireyi Z, et al. "Treatment is not yet necessary": delays in seeking access to HIV treatment in Uganda and Zimbabwe”. Afr J AIDS Res. 2018;17(3):217–225. 30132397 10.2989/16085906.2018.1490785

[17] Katz IT, Essien T, Marinda ET, et al. Antiretroviral therapy refusal among newly diagnosed HIV-infected adults. Aids. 2011;25(17):2177–2181. 21832935 10.1097/QAD.0b013e32834b6464 PMC3272300

[18] Larsen A, Cheyip M, Tesfay A, et al. Timing and predictors of initiation on antiretroviral therapy among newly-diagnosed HIV-infected persons in South Africa. AIDS Behav. 2019;23(2):375–385. 30008050 10.1007/s10461-018-2222-2 PMC6331268

[19] Ahmed S, Autrey J, Katz IT, et al. Why do people living with HIV not initiate treatment? A systematic review of qualitative evidence from low- and middle-income countries. Soc Sci Med. 2018;213:72–84. 30059900 10.1016/j.socscimed.2018.05.048 PMC6813776

[20] Lee MJ, Venturelli S, McKenna W, et al. Reasons for delayed antiretroviral therapy (ART) initiation in the era of early ART initiation guidelines: a retrospective service evaluation. Int J STD Aids. 2019;30(4):415–418. 30621548 10.1177/0956462418814985

[21] Kesselring S, Osborne C, Bever A, et al. Factors associated with delayed and late ART initiation among people living with HIV in BC: results from the engage study. AIDS Care. 2019;31(7):885–892. 30466303 10.1080/09540121.2018.1549722

[22] Musheke M, Bond V, Merten S. Deterrents to HIV-patient initiation of antiretroviral therapy in urban Lusaka, Zambia: a qualitative study. AIDS Patient Care Stds. 2013;27(4):231–241. 23530573 10.1089/apc.2012.0341

[23] Musheke M. Factors influencing uptake of HIV testing and non-initiation of and attrition from antiretroviral therapy care in Lusaka Zambia (Doctoral dissertation, University_of_Basel).

[24] Fox MP, Mazimba A, Seidenberg P, et al. Barriers to initiation of antiretroviral treatment in rural and urban areas of Zambia: a cross-sectional study of cost, stigma, and perceptions about ART. J Int AIDS Soc. 2010;13(1):8. 20205930 10.1186/1758-2652-13-8 PMC2847987

[25] Pry J, Chipungu J, Smith HJ, et al. Patient-reported reasons for declining same-day antiretroviral therapy initiation in routine HIV care settings in Lusaka, Zambia: results from a mixed-effects regression analysis. J Int AIDS Soc. 2020;23(7):e25560. 32618137 10.1002/jia2.25560 PMC7333172

[26] Sabapathy K, Mubekapi-Musadaidzwa C, Mulubwa C, et al. Predictors of timely linkage-to-ART within universal test and treat in the HPTN 071 (PopART) trial in Zambia and South Africa: findings from a nested case-control study. J Intern AIDS Soc. 2017;20(4):e25037. 10.1002/jia2.25037 PMC5810326 29251433

[27] Herce ME, Chi BH, Liao RC, et al. Re-thinking linkage to care in the era of universal test and treat: insights from implementation and behavioral science for achieving the second 90. AIDS Behav. 2019;23(Suppl 2):120–128. 31161462 10.1007/s10461-019-02541-5 PMC6773672

[28] Seeley J, Bond V, Yang B, et al. Understanding the time needed to link to care and start ART in seven HPTN 071 (PopART) study communities in Zambia and South Africa. AIDS Behav. 2019;23(4):929–946. 30415432 10.1007/s10461-018-2335-7 PMC6458981

[29] Pell C, Vernooij E, Masilela N, et al. False starts in 'test and start': a qualitative study of reasons for delayed antiretroviral therapy in Swaziland. Int Health. 2018;10(2):78–83. 29342259 10.1093/inthealth/ihx065

[30] Nhassengo P, Cataldo F, Magaço A, et al. Barriers and facilitators to the uptake of test and treat in Mozambique: a qualitative study on patient and provider perceptions. PLOS One. 2018;13(12):e0205919. 30586354 10.1371/journal.pone.0205919 PMC6306230

[31] Magaço A, Dovel K, Cataldo F, et al. 'Good health' as a barrier and facilitator to ART initiation: a qualitative study in the era of test-and-treat in Mozambique. Cult Health Sex. 2019;21(9):1059–1073. 30636559 10.1080/13691058.2018.1535091

[32] Layer EH, Kennedy CE, Beckham SW, et al. Multi-level factors affecting entry into and engagement in the HIV continuum of care in Iringa, Tanzania. PLOS One. 2014;9(8):e104961. 25119665 10.1371/journal.pone.0104961 PMC4138017

[33] Lieblich A, Tuval-Mashiach R, Zilber T. Narrative research: reading, analysis and interpretation. Bickman L, Rog DJ, editors. Thousand Oaks, California: Sage Publications; 1998.

[34] Wertz FJ, Charmaz K, McMullen LM, et al. Five ways of doing qualitative analysis. New York: The Guilford Press; 2011.

[35] Andrews M, Squire C, Tamboukou M, editors. Doing narrative research. London: Sage; 2013.

[36] Clandinin DJ, Connelly FM. Narrative inquiry: experience and story in qualitative research. San Francisco: John Wiley & Sons; 2004.

[37] Riessman CK. Narrative methods for the human sciences. London: Sage; 2008.

[38] Committee on Quality of Health Care in America. Crossing the quality chasm: a new health system for the 21st century. Washington, DC: Institute of Medicine, National Academies Press; 2001.

[39] Holmes CB. Estimated mortality on HIV treatment among active patients and patients lost to follow-up in 4 provinces of Zambia: findings from a multistage sampling-based survey. PLoS Med. 2018;15(1):e1002489. 10.1371/journal.pmed.1002489 PMC5766235 29329301

[40] Sikazwe I, Eshun-Wilson I, Sikombe K, et al. Retention and viral suppression in a cohort of HIV patients on antiretroviral therapy in Zambia: regionally representative estimates using a multistage-sampling-based approach. PLoS Med. 2019;16(5):e1002811. 31150380 10.1371/journal.pmed.1002811 PMC6544202

[41] Beres LK, Schwartz S, Simbeza S, McGready J, et al. Patterns and predictors of incident return to HIV care among traced, disengaged patients in Zambia: analysis of a prospective cohort. J Acquir Immune Defic Syndr. 2021;86(3):313–322. 33149000 10.1097/QAI.0000000000002554 PMC7878284

[42] Beres LK, Mwamba C, Bolton Moore C, et al. I cannot fail to come after people are worried about me’: factors and mechanisms enabling return to HIV care among previously disengaged patients in Zambia. AIDS Conference: Virtual. San Francisco, California, USA; 2020.

[43] Horter S, Wringe A, Thabede Z, et al. "Is it making any difference?" A qualitative study examining the treatment-taking experiences of asymptomatic people living with HIV in the context of Treat-all in Eswatini?”. J Int AIDS Soc. 2019;22(1):e25220. 30697970 10.1002/jia2.25220 PMC6351702

[44] Insight Start Study Group. Initiation of antiretroviral therapy in early asymptomatic HIV infection. New Eng J Med. 2015;373(9):795–807. 26192873 10.1056/NEJMoa1506816 PMC4569751

[45] Valverde-Villegas JM, de Medeiros RM, Ellwanger JH, et al. High CXCL10/IP-10 levels are a hallmark in the clinical evolution of HIV infection. Infection Genetics Evolution. 2018;57:51–58. 10.1016/j.meegid.2017.11.002 29122683

[46] Helova A, Akama E, Bukusi EA, et al. Health facility challenges to the provision of option B + in Western Kenya: a qualitative study. Health Policy Plan. 2017;32(2):283–291. 28207061 10.1093/heapol/czw122 PMC5886182

[47] Raveis VH, Siegel K, Gorey E. Factors associated with HIV-infected women's delay in seeking medical care. AIDS Care. 1998;10(5):549–562. 9828952 10.1080/09540129848415

[48] Gilbert L, Walker L. 'My biggest fear was that people would reject me once they knew my status.': stigma as experienced by patients in an HIV/AIDS clinic in Johannesburg, South Africa. Health Soc Care Community. 2010;18(2):139–146. 19708868 10.1111/j.1365-2524.2009.00881.x

[49] Kiwanuka J, Kiwanuka N, Kiweewa FM, et al. Factors associated with loss to follow-up in a primary healthcare HIV clinic practising test and treat. Journal of the international AIDS society. 2017. (Vol. 20, pp. 19–19). The Atrium, Southern Gate, Chichester po19 8sq, w Sussex, England: John Wiley & sons ltd.

[50] Katz IT, Dietrich J, Tshabalala G, Essien T, et al. Understanding treatment refusal among adults presenting for HIV-testing in Soweto, South Africa: a qualitative study. AIDS Behav. 2015;19(4):704–714. 25304330 10.1007/s10461-014-0920-y PMC4393756

[51] Dlamini-Simelane TT, Moyer E. ‘Lost to follow up’: rethinking delayed and interrupted HIV treatment among married Swazi women. Health Policy Plan. 2016;32(2):248–256. 10.1093/heapol/czw117 28207052

[52] Bor J, Chiu C, Ahmed S, et al. Failure to initiate HIV treatment in patients with high CD 4 counts: evidence from demographic surveillance in rural South Africa. Trop Med Int Health. 2018;23(2):206–220. 29160949 10.1111/tmi.13013 PMC5829292

[53] Horter S, Bernays S, Thabede Z, et al. "I don't want them to know": how stigma creates dilemmas for engagement with treat-all HIV care for people living with HIV in Eswatini”. Afr J AIDS Res. 2019;18(1):27–37. 30782082 10.2989/16085906.2018.1552163

[54] Mwamba C, Sharma A, Mukamba N, et al. They care rudely!’: resourcing and relational health system factors that influence retention in care for people living with HIV in Zambia. BMJ Glob Health. 2018;3(5):e001007. 10.1136/bmjgh-2018-001007 PMC6231098 30483408

[55] Zanolini A, Sikombe K, Sikazwe I, et al. Understanding preferences for HIV care and treatment in Zambia: evidence from a discrete choice experiment among patients who have been lost to follow-up. PLOS Med. 2018;15(8):e1002636. 30102693 10.1371/journal.pmed.1002636 PMC6089406

[56] Lilian RR, Rees K, McIntyre JA, Struthers HE, et al. Same-day antiretroviral therapy initiation for HIV-infected adults in South Africa: analysis of routine data. PLoS One. 2020;15(1):e0227572. 31935240 10.1371/journal.pone.0227572 PMC6959580

[57] Hailemariam TG, Nathan S, Seifu CN, et al. Uptake of couples HIV testing and counselling among heterosexual couples in Sub-Saharan Africa: a systematic review and meta-analysis. AIDS Care. 2020;32(2):137–147. 31116028 10.1080/09540121.2019.1619667

[58] Goldstein N, Pretorius HG, Stuart AD. The social construction of HIV/AIDS. Health SA Gesondheid. 2003;8(2):14–22.

[59] Barker C, Dutta A, Klein K. Can differentiated care models solve the crisis in HIV treatment financing? Analysis of prospects for 38 countries in Sub-Saharan Africa. J Int AIDS Soc. 2017;20(Suppl 4):21648. 28770597 10.7448/IAS.20.5.21648 PMC5577732

[60] World Health Organisation. Consultation on HIV differentiated service delivery models for specific populations and settings: pregnant and breastfeeding women, children, adolescents and key populations. Meeting report. Geneva, Switzerland; 2016.

